# Modeling of Catalytic Centers Formation Processes during Annealing of Multilayer Nanosized Metal Films for Carbon Nanotubes Growth

**DOI:** 10.3390/nano10030554

**Published:** 2020-03-19

**Authors:** Oleg I. Il’in, Nikolay N. Rudyk, Alexandr A. Fedotov, Marina V. Il’ina, Dmitriy I. Cherednichenko, Oleg A. Ageev

**Affiliations:** 1 Institute of Nanotechnologies, Electronics and Electronic Equipment Engineering, Southern Federal University, Taganrog 347922, Russia; mailina@sfedu.ru; 2 Laboratory of Nanobiotechnology Problems and New Materials, Southern Federal University, Taganrog, Taganrog 347922, Russia; nnrudyk@sfedu.ru (N.N.R.); aafedotov@sfedu.ru (A.A.F.); 3 Research and Education Center “Nanotechnologies”, Southern Federal University, Taganrog 347922, Russia; cherednichenko.sfedu@gmail.com (D.I.C.); ageev@sfedu.ru (O.A.A.)

**Keywords:** catalytic centers, thermal annealing, multilayer nanosized metal films, carbon nanotubes

## Abstract

The paper presents a theoretical model of the catalytic centers formation processes during annealing of multilayer nanosized metal films for carbon nanotubes growth. The approach to the description of the model is based on the mass transfer processes under the influence of mechanical thermoelastic stresses, which arise due to the difference in the thermal expansion coefficients of the substrate materials and nanosized metal layers. The thermal stress gradient resulting from annealing creates a drop in the chemical potential over the thickness of the film structure. This leads to the initiation of diffusion mass transfer between the inner and outer surfaces of the films. As a result, the outer surface begins to corrugate and fragment, creating separate islands, which serve as the basis for the catalytic centers formation. Experimental research on the formation of catalytic centers in the structure of Ni/Cr/Si was carried out. It is demonstrated that the proposed model allows to predict the geometric dimensions of the catalytic centers before growing carbon nanotubes. The results can be used to create micro- and nanoelectronics devices based on carbon nanotube arrays.

## 1. Introduction

Since the discovery of carbon nanotubes (CNTs) [1], their properties, not fully studied, have generated multiple research projects (since 1991 more than 135,000 articles have been published). The discovered unique properties [2,3,4] have shown promise for the use of CNTs as functional elements of emission nanoelectronic devices [5,6], memory elements [7,8], interconnects [9], as solar-driven water evaporation [10,11], generation of electricity [12], solar cells [13] and applications in the field of nanopiezotronics [14]. However, the creation of a broad range of CNTs-based devices requires their production with a given orientation relative to the substrate, sizes, density, properties and their location in areas determined by the device design [15].

A promising method for creating aligned CNTs is plasma enhanced chemical vapor deposition (PECVD) [16]. This method is catalytic and requires the formation of a transition metal thin film on a substrate. In the course of subsequent annealing, catalytic centers (CC) are formed of the thin film and, on their surface, the decomposition of carbon-containing gas molecules on the CC surface and can limit the further growth of CNTs. Thus, the parameters of the catalytic centers (size, dispersion, chemical composition, etc.) determine the parameters of carbon nanotubes (diameter, height, chirality, electrical properties, growth kinetics, etc.) that underlie their instrumental application. 

Aligned CNT arrays grown on a silicon substrate are often used for instrument applications [17,18]. To exclude chemical interaction between the substrate and the catalytic layer material (Ni, Fe, Co, etc.), as well as to create a metal CNTs contact, an intermediate layer is formed on the basis of metal films (Cr, V, Ti, etc.). During annealing, the intermediate metal layer of most metals used can interact with the silicon substrate forming the corresponding silicide.

Diffusion mass transfer is initiated during the heating of film structures between layers. As a result, the composition of the components changes in the layers volume and in the substrate contact area. The outer surface of the structure changes its profile. 

Diffusion exchange through the contact of a two-layer structure has been sufficiently studied [19,20]. However, the processes that occur during the creation of CC with controlled geometric parameters during annealing of the multilayer structure of nanoscale thickness metal films require additional studies.

Mechanical thermoelastic stresses have a significant effect on the nature of mass transfer processes and on the morphology of the multilayer structure during annealing [21]. These stresses arise due to the differences in the thermal expansion coefficients of the substrate materials and metal layers. In this case, the thermal stress gradient arising during heating creates a difference in the chemical potential over the thickness of the film structure. The difference in chemical potential causes diffusion mass transfer between the inner and outer surfaces of the multilayer structure. Since the inner surface of the film is blocked by a layer of the formed metal silicide of the sublayer, the possibility of its free expansion is excluded. Due to the escape of diffusing atoms on the surface, the outer surface of the structure begins to corrugate. Subsequently, the corrugated profile can fragment forming separate islands which are the basis for the CC formation on the substrate surface.

Thus, taking into account the influence of mass transfer processes and the formation of thermoelastic stresses on the morphology of the film structure in the course of heating will allow us to determine the laws of shaping in a multilayer structure. The established laws can be used to generate carbon nanotubes catalytic centers growth with controlled parameters.

The aim of this work is to develop a model of thermophysical processes of the catalytic centers formation for the carbon nanotubes growth during annealing of nanoscale thickness metal films on a silicon substrate and its experimental approbation for the Ni/Cr/Si structure.

## 2. Thermal Stresses in the Structure in the Course of Annealing

With increasing temperature at the initial stage of heating, a superposition of two stresses arises in the structure:

(1) $$\sigma =\sigma_{\mathrm{Dyn}}+\sigma_{\mathrm{Int}},$$

where dynamic thermoelastic stress (*σ_Dyn_*) is determined by the rate of temperature increase at the heating stage:

(2) $$\sigma_{\mathrm{Dyn}}\left(t\right)=\frac{\alpha \times E\times h^{2}}{\left(1-\upsilon \right)\times a}\frac{\mathrm{dT}}{\mathrm{dt}}=\frac{\alpha \times E\times h^{2}}{\left(1-\upsilon \right)\times a}\cdot \frac{T}{\tau }\times \exp\left(-\frac{t}{\tau }\right),$$

and stress arising at the metal/substrate interface (*σ_Int_*) depends on the difference in thermal expansion coefficients of materials [22]:

(3) $$\;\;\sigma_{\mathrm{Int}\;}\left(t\right)=\left(\alpha_{\mathrm{sub}}{}_{\;}-\alpha_{\mathrm{sl}}-\alpha_{\mathrm{cl}}\right)\times E\times T\left(t\right),$$

where α is a coefficient of the sublayer material thermal expansion, *h*—metal layer thickness, *a*—thermal diffusivity, *T*—temperature, τ—parameter that determines the rate of heating of the structure, *t*—time, *E*—Young’s modulus for metal (sublayer), *υ*—Poisson’s ratio of the substrate material, *α_sub_,* *α_sl_,α_cl_*—thermal expansion coefficients of the substrate material, sublayer and catalytic layer, respectively.

As follows from Equation (2), σ*_Dyn_* has a maximum value at the initial stage of heating, therefore, the appearance of this stress can cause cracking of the film in a still unheated structure.

## 3. Mass Transfer in the Course of Annealing

A multilayer structure, consisting of two or more layers of different materials, is initially thermodynamically non equilibrium, so there is a continuous diffusion exchange of atoms between the layers. In accordance with the principles of thermodynamics, the dominant diffusion flow is directed from the layer with a higher latent heat of evaporation, because only in this case the free energy change of the system will be maximum [20]. Since silicon has the highest heat of vaporization in the Ni/Cr/Si model structure, the flow of silicon atoms through the metal layers will be predominant. On the outer surface of a metal film with h thickness (where the total thickness of the catalytic layer and sublayer does not exceed 50 nm), the migrating silicon atom can escape and sublimate into vacuum, making *h/(2·a)* jumps through the points of the lattice crystalline metal film. In this case, the lifetime of the migrating atom in the point of the lattice: $\tau_{d}≅a^{2}/D$, where *D* is a diffusion coefficient of silicon.

At temperatures above ~450 °C, chromium atoms will penetrate the silicon substrate within the diffusion zone. A layer of chromium silicide will be formed on the inner surface of the film, fixing the metal film relative to the silicon substrate. Therefore, in accordance with the thermal expansion coefficients of the layers and the substrate, the metal/substrate interfacial plane will be in a compression state. The resulting difference in chemical potential (Δμ) will cause diffusion transfer of matter between inner and outer surface of the structure:

(4) $$∆\mu =\left(\sigma_{\mathrm{Int}}-2\times \gamma_{\mathrm{out}}\times K\right)\times \Omega ,$$

where *γ_out_* is a specific surface energy of the outer layer, *K*—surface curvature, *Ω*—atom volume of the outer layer material.

As a result of the inflow of material, the outer surface of the metal film will deform. Therefore, when equilibrium is reached, the condition Δ*μ* = 0 is satisfied. The curvature of the equilibrium profile element will be determined by the equality of the elastic stress forces and capillary pressure:

(5) $$K=\frac{1}{R_{m}}=\frac{\sigma_{\mathrm{Int}}}{2\times \gamma_{\mathrm{out}}},$$

where *R*_m_ is the radius of the profile element curvature.

The value of the specific surface energy (*γ*) of the outer and inner metal layers in the solid state of aggregation was estimated by the formula:

*γ* = *E*x*a*/2 (6)

Representing the surface curvature of the equilibrium profile element through the contact angle (*φ*):

(7) $$K=-\frac{d\varphi }{\mathrm{ds}\;}=-\frac{d\varphi }{\mathrm{dx}}\times \sin\varphi =-\frac{d\varphi }{\mathrm{dy}}\times \cos\varphi ,$$

it is possible to evaluate the height and the radius of the profile element base on the outer surface of the metal as a function of the contact angle φ:

(8) $$\;h_{*}=R_{m}\times \left(1-\cos\varphi \right),$$

(9) $$R=R_{m}\times \sin\varphi .$$

In the course of the profile element formation along its base on the outer surface of the structure the recess is formed. In this case, the outer layer maintains integrity as long as the depth of the recess remains less than the thickness of the layer. On the basis of the equalities (8)–(9), we can obtain an equation for determining the contact angle corresponding to the break moment of the metal layer:

(10) $$\left[\frac{h_{0}}{R_{m}}-(1-\cos\left(\varphi_{*}\right)\right]=0,$$

where *h*_0_ is thickness of the outer metal layer.

After the breaking of the upper layer, when the profile of the isolated element approaches the equilibrium configuration as a result of relaxation, the contact angle will vary within $\varphi_{*\;}\leq \;\varphi \leq \varphi_{k}$ (where ϕ_*_ is a contact angle of the outer layer breaking, *ϕ*_k_—contact angle of the CC stationary profile).

## 4. The Formation of Catalytic Centers

Isolated profile elements formed as a result of film breaking are nuclei of catalytic centers. According to the kinetic theory of the initial stage of phase transformations [23], even the stable, supercritical centers of the new phase, due to their small size $R\leq 10\times R_{k}$ (where *R_k_* is a critical radius) will not be faceted in accordance with the crystallographic parameters of the material. Therefore, we can assume that the isolated profile elements created during the film breaking will be formed as spherical segments. Since the geometric dimensions of the catalytic centers are nanoscale objects, its shape factor (SF) will have a significant effect on the dynamics of formation and the final profile of the CC. After the film ruptures, the dominant factor controlling the formation of the CC profile is the specific surface energy. Thermal stress only supports the transfer of matter through the film fragment, on which the developing CC is based.

The numerical value of the shape factor (δ) of a geometric object is equal to the ratio of the surface area to its volume. It can be expected that the SF of CC with the profile of the spherical segment of nanoscale radius *R* will have an extremely large value [23]:

(11) $${\delta (\varphi ,\;R)|}_{\varphi_{*}}=\frac{S}{V}=\frac{6}{R\times \left(1-\cos(\varphi )\right)\times \left(2+\cos\left(\varphi \right)\right)}\gg 1,$$

where *S* is the area of the outer surface of the spherical segment, *V*—the spherical segment volume.

Before the film breaking, we can consider CC as a bulge on the structure surface. However, when the outer layer breaks, the surface of the sublayer is opened. As a result, we can observe a change in the nature of the interaction of the forces acting on the surface and on the perimeter of the base of the CC:

(12) $$\Delta µ=\left[\sigma_{\mathrm{Int}}-\frac{2\gamma_{\mathrm{out}}}{R}\left(1-\mathrm{ctg}\left(\varphi \right)+\frac{\gamma_{\mathrm{in}}}{\gamma_{\mathrm{out}}}\times {\left(\sin(\varphi )\right)}^{-1}\right)\right]\times \Omega ,$$

where *γ*_in_ is a specific surface energy of the inner layer.

Since at Δ*µ* = 0 the radius and the contact angle of the CC remain unknown, the Equation (13) establishes only the functional relationship between the characteristic parameters of the equilibrium profile of the CC:

(13) $$R=R_{m}\times \left[1-\mathrm{ctg}(\varphi )+\frac{\gamma_{\mathrm{in}}}{\gamma_{\mathrm{out}\;}}\times {\left(\sin(\varphi )\right)}^{-1}\right]$$

To use this relation in future, it is necessary to identify additional physical correlations between the radius and the contact angle of the CC. It is possible to compose a transcendental equation to determine the contact angle of the stationary profile of the CC, if you notice that the product of the surface tension forces (specific surface energy) of the outer layer on the shape factor of the stationary CC is equal to the acting effective thermoelastic stress:

(14) $$\sigma_{\mathrm{Int}}=2\times \gamma_{\mathrm{out}\;}\times \delta \left(\varphi_{k}\right),$$

where

(15) $$\delta (\varphi_{k})=\frac{6}{\;R\left(\varphi_{k}\right)\times \left(1-\cos(\varphi_{k}\right)\times \left(2+\cos(\varphi_{k}\right))}$$

## 5. Evolution of the Catalytic Center Profile

Having determined the gradient of the chemical potential as ∇*μ* = Δ*μ/h*_0_, we can demonstrate that the intensity of the diffusion flow (*F*) to the base of the CC will be determined as:

(16) $$F\left(\varphi \right)=D\times \frac{n_{0}}{kT}\times \frac{\Omega }{h_{0}}\times \sigma_{0}\left(1-\frac{R_{k}}{R\left(\varphi \right)}\right),$$

where *n*_0_ = *ρ·N_A_*x*M*^−1^ is a concentration of atoms in the lattice of the outer layer material, *ρ*—density of the outer layer material; *M*—atomic weight of the outer layer material; *N_A_*—Avogadro number, *k*—Boltzmann constant.

Considering the loss of material due to sublimation of atoms from the surface of CC, we can formulate the equation for the evolution of its profile to a stationary configuration:

(17) $$\frac{\mathrm{dR}}{\mathrm{dt}}=\beta_{D}\left(1-\frac{R_{k}}{R}\right)-\beta_{C}\left(1+\chi \frac{R_{k}}{R}\right),$$

where the kinetic parameters

(18) $$\beta_{D}=D\times \frac{n_{0}}{\mathrm{kT}}\times \frac{\Omega^{2}}{h_{0}}\times \sigma_{\mathrm{Int}},$$

(19) $$\beta_{C}=\frac{P_{0}\times \Omega }{{\left(2\pi \times m\times k\times T\right)}^{\frac{1}{2}}}$$

determine diffusion transport (*β_D_*) and substance loss rate (*β_C_*) upon sublimation of atoms from the outer surface of the CC, respectively; *P_0_* is an equilibrium pressure of saturated vapor of the outer layer material above a flat surface at annealing temperature [24]; $\;\chi =\frac{\sigma_{0}\times \Omega }{\mathrm{kT}}$ = 5.339 × 10^−8^ – dimensionless energy factor, *m*—molar mass.

The process of evolution of the CC profile can be traced by the rate of the contact angle changing. To do this, changing the order of differentiation and taking into account the dependence of the radius CC on the contact angle value (13), the Equation (17) can be converted into:

(20) $$-\frac{d\varphi }{\mathrm{dt}}=\theta_{1}\left(1-\frac{\theta }{\theta_{1}}\times \frac{R_{k}}{R}\right)\times {\left(\frac{\mathrm{dR}}{d\varphi }\right)}^{-1},$$

where *θ* = (*β_D_ + χxβ_C_·*) and *θ*_1_ = (*β_D_ − β_C_*).

By integrating the Equation (20) within the limits of the change in the contact angle (*ϕ*) from the layer rupture moment (ϕ_*_) to the CC steady-state profile formation moment (*ϕ_k_*), we can determine the time of its formation:

(21) $$t_{k}=\frac{1}{\theta_{1}}\times \int_{\varphi *}^{\varphi k}\frac{d\varphi }{\left(1-\frac{\theta }{\theta_{1}}\times \frac{R_{k}}{R}\right)\times {\left(\frac{\mathrm{dR}}{d\varphi }\right)}^{-1}}\;,$$

where *t_k_* is an estimated time of formation of a CC of a radius *R_k_(ϕ_k_)*.

The Equation (20) demonstrates that the CC relaxation process is quasi-stationary, since at the moment when the radius of the CC profile approaches its limiting value *R = R_k_*, only the matter flow to the CC base is blocked, while the sublimation process at a constant annealing temperature will continue until the center disappears completely. The equation of the CC relaxation process at this stage of annealing will have the form of:

(22) $$\frac{\mathrm{dR}}{\mathrm{dt}}=-\beta_{C}\left(1+\chi \frac{R_{k}}{R}\right)$$

Integrating the Equation (22) under the initial condition ${R|}_{t=0}$= *R*_k_, we can find the time during which the CC completely sublimates:

(23) $$t_{k}=\frac{R_{k}}{\beta_{C}}\left(1-\chi \times \ln\left(\frac{1+\chi }{\chi }\right)\right).$$

## 6. Experiments and Methods

Experimental studies of the laws of catalytic centers formation on the basis of a continuous film were carried out using the PECVD equipment (NT-MDT, Zelenograd, Russia). Chemically purified Si (100) chips 8 × 8 mm we used as substrates. Cr and Ni metal films with a thickness of 20 nm and 10 nm, respectively, were deposited by magnetron sputtering chromium (99.95% purity, Kurt J. Lesker Co., Jefferson Hills, PA, USA) and nickel targets (99.995% purity, Kurt J. Lesker Co., Jefferson Hills, PA, USA) on AUTO500 (BOC Edwards, Burgess Hill,UK) at a pressure of 83.1 Pa. 

**Figure 1:**
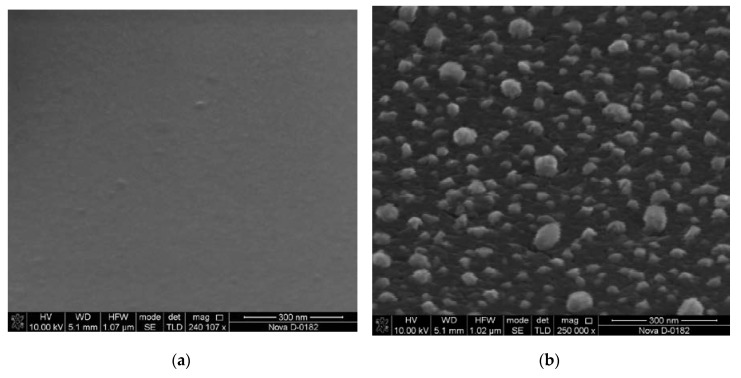
SEM image of film before heating (**a**) and catalytic centers (CC) obtained at a heating temperature of 750 °C (**b**).

We studied the influence of the annealing temperature on the formation of catalytic centers for the created samples with the Ni/Cr/Si structure. The heating was carried out in a temperature range of 700–800 °C for 20 min in argon and ammonia flows (40 and 15 sccm, respectively). The geometric parameters of the formed CC were studied by atomic force microscopy (AFM) in the semi-contact mode, using the Ntegra (NT-MDT, Zelenograd, Russia) and scanning electron microscopy (SEM) using the Nova Nanolab 600 (FEI, Hillsborough, OR, USA). Analysis of the AFM and SEM images showed that the surface roughness of the samples with the Ni/Cr/Si structure was 2 ± 0.5 nm. After heating the substrate to a predetermined temperature, the metal film fragmented with the formation of catalytic centers (Figure 1).

The diameter and height of the CC (Table 1) were determined by statistical processing of the obtained AFM images using the Image Analysis software package.

## 7. Results and Discussion

According to the observed experimental laws, the heating kinetics can be approximated by the dependence:

(24) $$T\left(t\right)=T_{0}\left(1-e^{-\frac{t}{\tau }}\right)\;,$$

where *τ* is a characteristic parameter, *T*_0_ – predetermined heating temperature.

Assuming that the heating stage is completed at *T ≥* 0.99 × *T*_0_, the characteristic parameter can be estimated by the formula:

(25) $$\tau =\frac{t}{\ln\left(\xi \right)}\;,$$

where ξ is a parameter determining the heating rate (under the condition *T* = 0.99 × *T*_0_, ξ = 100).

An analysis of the heating rate of real equipment used in experimental studies showed their good agreement with the approximate dependence (27) at a level of 95%. The values of the physical quantities used in the numerical simulation are presented in Table 2.

**Figure 2:**
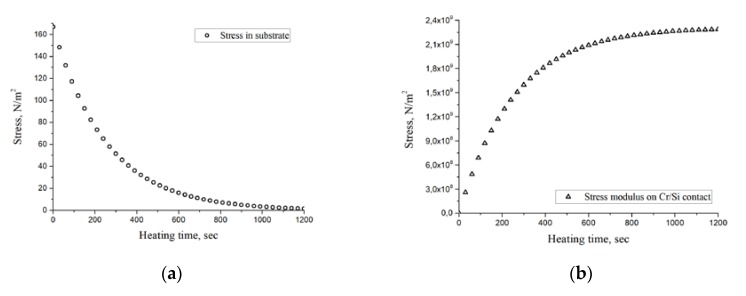
The dependence of stress on time arising in the substrate (**a**) and in the contact plane (**b**).

**Figure 3:**
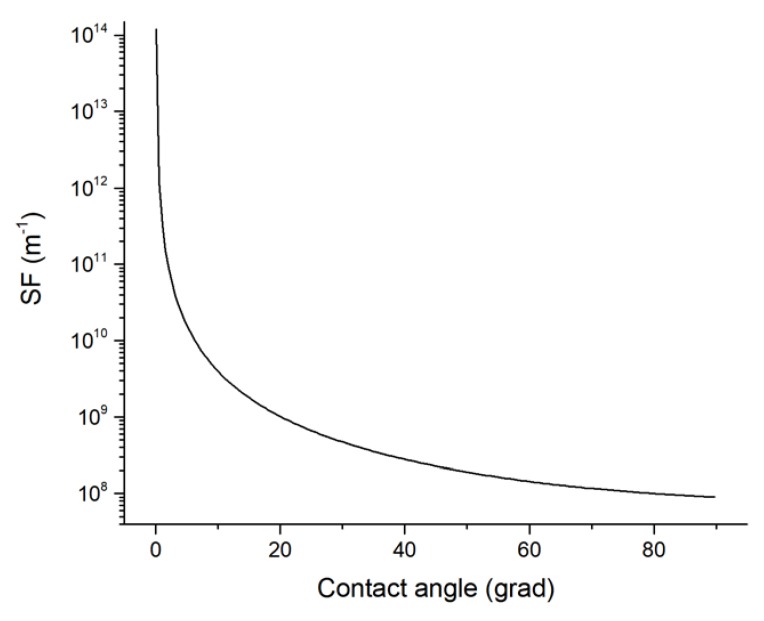
Simulation of the change in shape factor (SF) on the contact angle.

Calculations by Formula (2) showed that at the heating temperature of 750 °C, the thickness of the structure (including the substrate) of 5 × 10^−4^ m and the initial heating rate of 3 deg/s, the dynamic thermoelastic stresses are 160 Pa (Figure 2a), which is significantly less than the tensile strength of silicon substrate. During the heating time (t* = 1200 s), dynamic thermoelastic stresses decrease almost to zero. In this case, the stress arising at the metal/substrate interface *σ_Int_(t)* continuously increases with increasing temperature according to (2), (25) and after reaching the stationary heating mode remains constant *σ_Int_* = 2.2 × 10^9^ Pa (Figure 2b). The dependence of the change in SF on the contact angle in accordance with Equation (11) is shown in Figure 3.

After the solution of the Equation (10) we obtained the value of the contact angle of 1.1609 rad (66.5°), that corresponds to a layer breaking with a thickness *h_0_* = 20 × 10^−9^ m and the formation of CC with a height of h* = 2.001 × 10^−8^ m and a radius of the base R* = 3.052 × 10^−8^ m. Moreover, the estimation of the CC shape factor with the profile of the spherical segment of radius R_*_ was δ(ϕ_*_,$R_{*}$) = 1.25 × 10^8^ m^−1^.

A numerical estimated value of the contact angle satisfying the Equation (14) was *φ_к_* = 1.555 rad (89°). Based on this, using Formulas (8), (9) and (13), we calculated the radius of the stationary CC profile *R(φ_k_)* = 1.006 × 10^−7^ m, diameter of its base (2.012 × 10^−7^ m) and its height *h_k_* = 9.903 × 10^−8^ m. The shape factor in accordance with (15) *δ*(ϕ_k_) = 9.089 × 10^7^ m^−1^.

**Figure 4:**
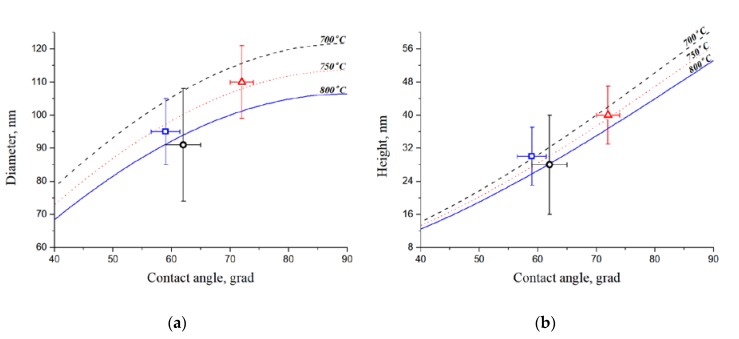
Dependences of the diameter (**a**) and height (**b**) of the catalytic centers on the contact angle obtained at different temperatures.

The analysis of results of experimental samples research by the AFM allowed us to obtain the CC contact angles values, which correspond to the theoretical laws (Figure 4) calculated on the basis of the proposed model.

## 8. Conclusions

The paper presents a theoretical model of the thermophysical processes of the catalytic centers formation for the carbon nanotubes growth by annealing of metal films on a silicon substrate. It was discovered that the difference between the temperature expansion coefficients of the Si substrate and the Cr sublayer film contributes to the appearance of strong mechanical stresses in the film/substrate contact area in the course of heating. This results in fragmentation and rupture of the metal film into individual islands.

The obtained experimental results demonstrate that the heating temperature of the structure Ni (10 nm)/Cr (20 nm)/Si (380 μm) affects the geometric parameters of the formed catalytic centers. It was shown that the beginning of the catalyst film rupture process (700 °C) is accompanied by a high dispersion of the CC diameter and height due to weak surface diffusion [27]. At a heating temperature of 750 °C, the merger of small CCs by larger ones is observed due to the continuous process of mutual diffusion between the layers of the structure [27]. With an increase in the annealing temperature above 750 °C, the processes of thermal etching and surface diffusion occur, which leads to a decrease in the catalytic centers diameter and height with a decrease in the quantity of diminutive CC. The dependence of the CC diameter and height on the contact angle is shown at annealing temperatures of 700–800 °C.

The proposed model can be used to predict the size of CC with the spherical shape during annealing of multilayer metal films and can be used to develop technological processes of the carbon nanotubes formation for vacuum microelectronics, microelectronic sensors, nano- and micro-systems, and nanoelectronics.

## Figures and Tables

**Table 1:** Geometric parameters of catalytic centers.

Parameters of Catalytic Centers	Heating Temperature, °C		
	700	750	800
Diameter, nm	91 ± 17	110 ± 11	95 ± 10
Height, nm	28 ± 12	40 ± 7	30 ± 7

**Table 2:** Parameters used in numerical simulation.

Physical Quantity	Estimation Formula	Si	Cr	Ni	Units	Ref.
Thermal conductivity, λ	constant	36.5	–	–	$\frac{J}{s\times m\times {}^{\circ}K}$	[25]
Density, ρ	constant	2.33 × 10^3^	7.1 × 10^3^	8.75 × 10^3^	$\frac{kg}{m^{3}}$	[25]
Molar weight, *M*	constant	28.06 × 10^−3^	51.99 × 10^−3^	58.71 × 10^−3^	$\frac{kg}{mol}$	[25]
Heat capacity, *c*	constant	19.79	–	–	$\frac{J}{{}^{\circ}K}$	[25]
Thermal diffusivity, *a_TD_*	$a_{TD}=\frac{\lambda }{\rho \times c}$	7.92 × 10^4^	–	–	$\frac{m^{2}}{s}$	
Atom concentration in the lattice, *N*	$N=\frac{\rho }{M}\times N_{A}$	5 × 10^28^	8.22 × 10^28^	8.98 × 10^28^	$m^{-3}$	
Atom volume, *Ω*	$\Omega =\frac{1}{N}$	2 × 10^−29^	1.22 × 10^−29^	1.11 × 10^−29^	$m^{3}$	
Atom radius, *r*	constant	1.68 × 10^−10^	1.7 × 10^−10^	1.24 × 10^−10^	m	[25]
Atom diameter, *a*	a=2×r	3.36 × 10^−10^	3.4 × 10^−10^	2.48 × 10^−10^	m	
Young’s modulus, *E*	constant	110 × 10^9^	297 × 10^9^	200 × 10^9^	$\frac{N}{m^{2}}$	[26]
Poisson’s ratio, *υ*	constant	0.288	0.21	0.3	–	[25]
Coefficient of thermal expansion, *α*	constant	4.65	10.98	18.2	$\frac{10^{-6}}{{}^{\circ}K}$	[26]
Specific surface energy, *γ*	$\gamma =\frac{E\times a}{2}$	18.52	50.58	24.8	$\frac{N}{m}$	[25]

## References

[1] Iijima S. (1991). Helical microtubules of graphitic carbon. Nature.

[2] Ebbesen T.W., Lezec H.J., Hiura H., Bennett J.W., Ghaemi H.F., Thio T. (1996). Electrical conductivity of individual carbon nanotubes. Nature.

[3] Treacy M.M.J., Ebbesen T.W., Gibson J.M. (1996). Exceptionally high Young’s modulus observed for individual carbon nanotubes. Nature.

[4] Il’ina M.V., Il’in O.I., Blinov Y.F., Smirnov V.A., Kolomiytsev A.S., Fedotov A.A., Konoplev B.G., Ageev O.A. (2017). Memristive switching mechanism of vertically aligned carbon nanotubes. Carbon.

[5] Lim Y.D., Kong Q., Wang S., Tan C.W., Tay B.K., Aditya S. (2019). Enhanced field emission properties of carbon nanotube films using densification technique. Appl. Surf. Sci..

[6] Chen Z., Cao G., Zhang Q., Lan P., Zhu B., Yu T., Lin Z. (2007). Large current carbon nanotube emitter growth using nickel as a buffer layer. Nanotechnology.

[7] Ageev O.A., Blinov Y.F., Il’in O.I., Konoplev B.G., Rubashkina M.V., Smirnov V.A., Fedotov A.A. (2015). Study of the resistive switching of vertically aligned carbon nanotubes by scanning tunneling microscopy. Phys. Solid State.

[8] Tsai C.-L., Xiong F., Pop E., Shim M. (2013). Resistive Random Access Memory Enabled by Carbon Nanotube Crossbar Electrodes. ACS Nano.

[9] Srivastava A., Liu X.H., Banadaki Y.M. (2017). Overview of Carbon Nanotube Interconnects. Carbon Nanotubes for Interconnects.

[10] Dao V.-D., Vu N.H., Yun S. (2020). Recent advances and challenges for solar-driven water evaporation system toward applications. Nano Energy.

[11] Dao V.-D., Choi H.-S. (2018). Carbon-Based Sunlight Absorbers in Solar-Driven Steam Generation Devices. Glob. Chall..

[12] Dao V.-D., Vu N.H., Choi H.-S. (2020). All day Limnobium laevigatum inspired nanogenerator self-driven via water evaporation. J. Power Sources.

[13] Dang H.-L.T., Tran N.A., Dao V.-D., Vu N.H., Quang D.V., Vu H.H.T., Nguyen T.H., Pham T.-D., Hoang X.-C., Nguyen H.T. (2020). Carbon nanotubes-ruthenium as an outstanding catalyst for triiodide ions reduction. Synth. Metals.

[14] Il’ina M., Il’in O., Blinov Y., Konshin A., Konoplev B., Ageev O. (2018). Piezoelectric Response of Multi-Walled Carbon Nanotubes. Materials.

[15] Mitra M. (2019). Characteristics of Carbon Nanotube Relay. Sci. J. Res. Rev..

[16] Liu J., Jiang D., Fu Y., Wang T. (2013). Carbon nanotubes for electronics manufacturing and packaging: From growth to integration. Adv. Manuf..

[17] Gupta B.K., Kedawat G., Gangwar A.K., Nagpal K., Kashyap P.K., Srivastava S., Singh S., Kumar P., Suryawanshi S.R., Seo D.M. (2018). High-performance field emission device utilizing vertically aligned carbon nanotubes-based pillar architectures. AIP Adv..

[18] Klinke C. (2003). Analysis of Catalytic Growth of Carbon.

[19] Cherepanov G.P. (1997). Physics of Sintering. Methods of Fracture Mechanics: Solid Matter Physics.

[20] Pines B.Y., Geguzin Y.E. (1953). Self-diffusion and heterodiffusion in heterogeneous porous bodies. J. Tech. Phys..

[21] Antman S.S. (1995). Nonlinear Problems of Elasticity.

[22] Mullins W.W. (1961). Theory of linear facet growth during thermal etching. Philos. Mag..

[23] Bello I. (2017). Vacuum and Ultravacuum: Physics and Technology.

[24] Frenkel J. (1946). Kinetic Theory of Liquids.

[25] Kikoin I. (1976). Tables of Physical Constants.

[26] Grigoriev I., Meilikhov E., Radzig A. (1996). Handbook of Physical Quantities.

[27] Il’in O.I., Rudyk N.N., Il’ina M.V., Osotova O.I., Fedotov A.A. (2019). Influence of annealing temperature and activation time on the catalytic centers formation for carbon nanostructures growth. J. Phys. Conf. Ser..

